# 1879. Paradoxical Reaction in Lymph Node Tuberculosis among HIV-negative Patients: Incidence, Predictive Factors, and Clinical Outcomes

**DOI:** 10.1093/ofid/ofad500.1707

**Published:** 2023-11-27

**Authors:** Ankesh Gupta, Santosh Kumar, Pankaj Jorwal, Binit K Singh

**Affiliations:** All India Institute of Medical Sciences, New Delhi, New Delhi, Delhi, India; All India Institute of Medical Sciences, Delhi, Delhi, India; All India Institute of Medical Sciences, Delhi, Delhi, India; All India Institute of Medical Sciences, Delhi, Delhi, India

## Abstract

**Background:**

The aim of this study is to evaluate the occurrence of paradoxical reactions (PRs), cytokine levels and outcome in HIV-negative lymphnode tuberculosis (TB) patients.

**Methods:**

This is a prospective observational study conducted at the All-India Institute of Medical Sciences in New Delhi, India from July 2019 to June 2021. 144 subjects with confirmed or probable lymph node TB, excluding MDR TB, were recruited. Baseline characteristics, clinical presentation, imaging, laboratory parameters, and TB diagnostic modalities were gathered. Patients were followed up to evaluate the occurrence of PRs, and cytokines IL2, IL6, IL18, and TNF alpha were analyzed using the sandwich ELISA principle at baseline and after PR development.

**Figure:**
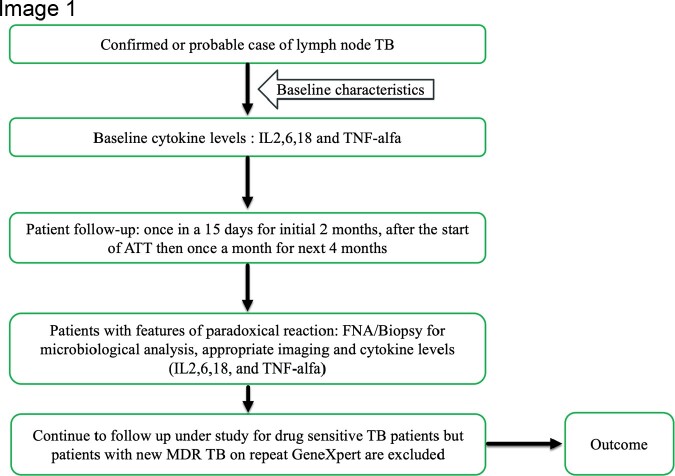

Workflow of the study.

**Results:**

Total 144 subjects with confirmed tuberculosis were recruited. PRs developed in 5.5% of patients, with fever (58.74%) and weight loss (44.06%) being the predominant systemic symptoms. The median time for PR development was 37 days, with pain and swelling being the most common presentation (62.5%). Among the baseline cytokines, IL6 was the most elevated (20.83%), with IL2, IL18, and TNF alpha being elevated in 8.3%, 0.69%, and 1.38% of the subjects, respectively. On multivariate analysis, baseline elevation of interleukin 2 was statistically significant and predicted PRs. IL2 and IL6 were significantly elevated during the occurrence of PRs, while IL18 and TNF alpha were not. Most patients (75%) improved on NSAIDs, with a median symptom resolution duration of 30 days.

**Figure:**
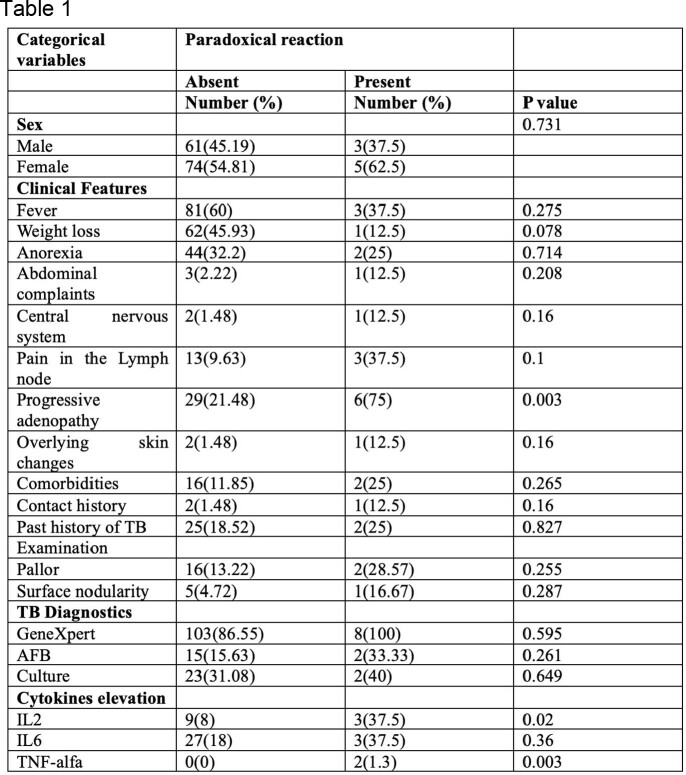

Comparison of patient parameters at baseline and after the development of paradoxical reactions.

**Figure:**
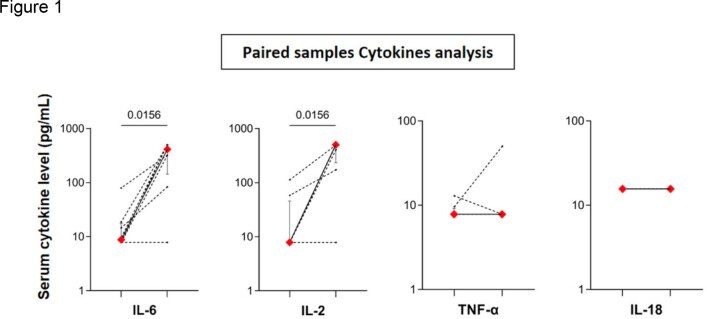

Comparison of cytokine levels at baseline and after paradoxical reactions.

**Figure:**
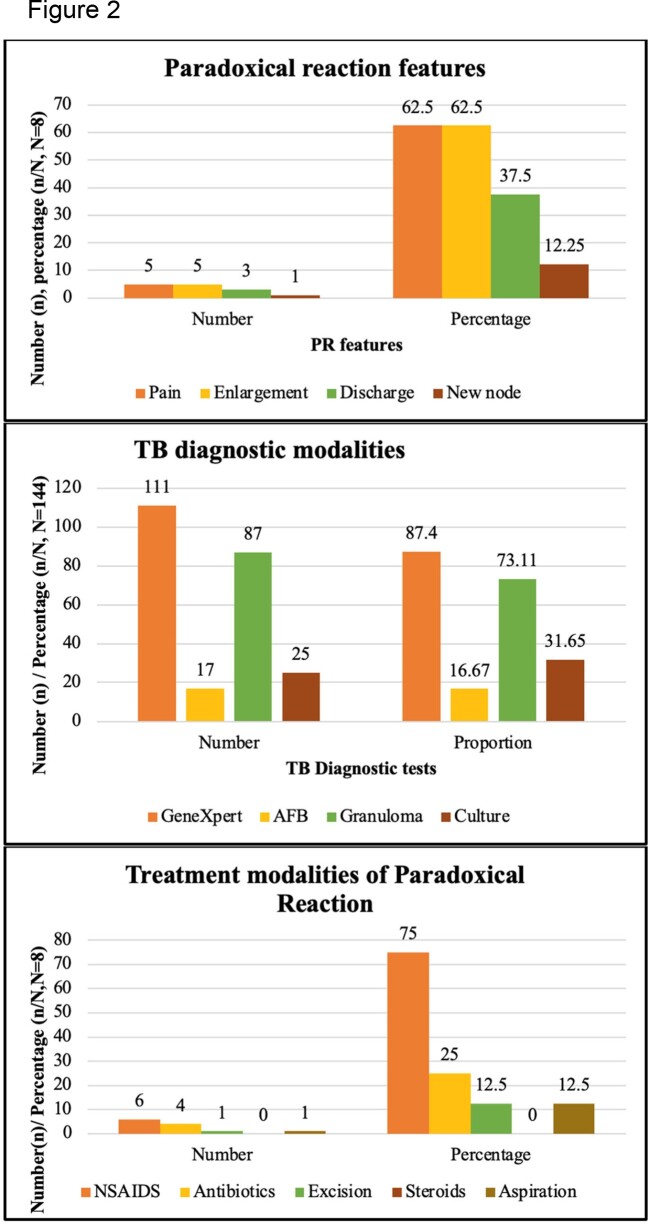

Paradoxical reaction features, tuberculosis diagnostic modalities and treatment modalities of paradoxical reaction.

**Conclusion:**

Our study found that baseline elevation of IL-2 and worsening adenopathy and weight loss during treatment are potential predictors for paradoxical reaction in lymph-node tuberculosis. However, it is important to validate these findings through larger prospective studies.

**Disclosures:**

**All Authors**: No reported disclosures

